# Tautomerisation Mechanisms in the Adenine-Thymine Nucleobase Pair during DNA Strand Separation

**DOI:** 10.1021/acs.jpcb.2c08631

**Published:** 2023-03-20

**Authors:** Benjamin King, Max Winokan, Paul Stevenson, Jim Al-Khalili, Louie Slocombe, Marco Sacchi

**Affiliations:** † Department of Physics, University of Surrey, Guildford GU2 7XH, U.K.; ‡ Leverhulme Quantum Biology Doctoral Training Centre, University of Surrey, Guildford GU2 7XH, U.K.; ¶ School of Chemistry and Chemical Engineering, University of Surrey, Guildford GU2 7XH, U.K.

## Abstract

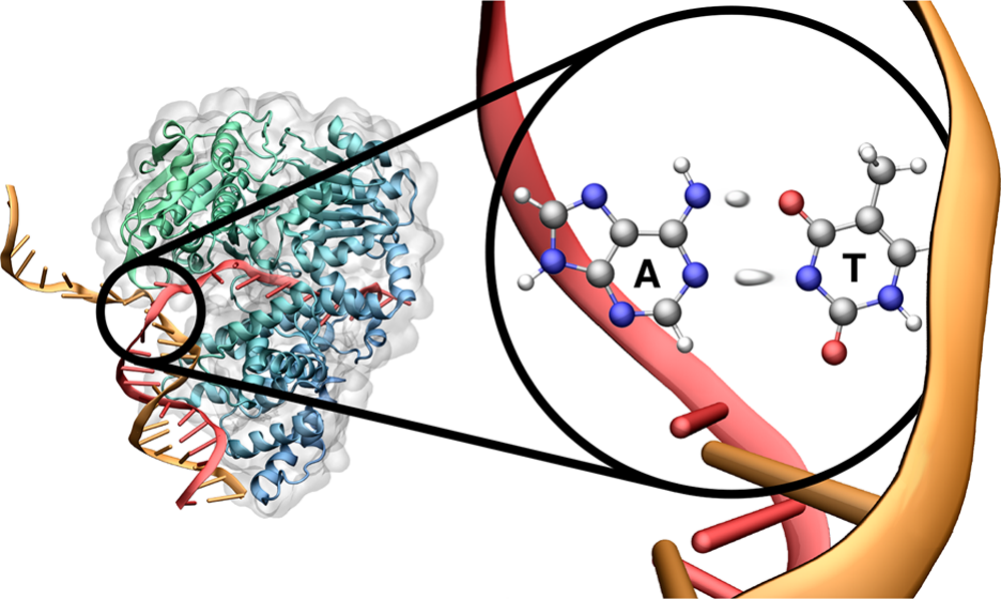

The adenine-thymine tautomer (A*-T*) has previously been discounted as a spontaneous mutagenesis mechanism due to the energetic instability of the tautomeric configuration. We study the stability of A*-T* while the nucleobases undergo DNA strand separation. Our calculations indicate an increase in the stability of A*-T* as the DNA strands unzip and the hydrogen bonds between the bases stretch. Molecular Dynamics simulations reveal the time scales and dynamics of DNA strand separation and the statistical ensemble of opening angles present in a biological environment. Our results demonstrate that the unwinding of DNA, an inherently out-of-equilibrium process facilitated by helicase, will change the energy landscape of the adenine-thymine tautomerization reaction. We propose that DNA strand separation allows the stable tautomerization of adenine-thymine, providing a feasible pathway for genetic point mutations via proton transfer between the A-T bases.

## Introduction

**Figure 1:**
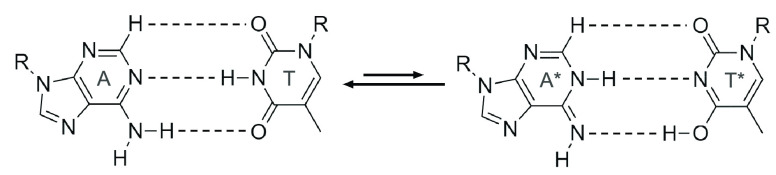
Tautomerization mechanism for the A-T base pair. A = adenine, T = thymine, A* = the tautomer of adenine, and T* = the tautomer of thymine. Here, “R” indicates where the base binds to the sugar–phosphate backbone of the DNA strand. The dashed lines represent the hydrogen bonds between the base pairs. The forward–backward arrow indicates the reversibility of the process, with the larger backward arrow indicating that the canonical configuration is more stable and preferred energetically.

Spontaneous mutagenesis describes how the genetic code of DNA can incorporate errors without the influence of external factors. Generally, these errors disrupt the canonical (or Watson–Crick) base pairings, adenine-thymine (A-T) and guanine-cytosine (G-C). The root idea that the tautomerization of DNA may be a mechanism promoting genetic mutation dates back to a short, tentative hypothesis from Watson and Crick’s second paper of 1953, where the process of mitosis (the self-replication of DNA) was first theorized.^1^ Since then, many studies have investigated the validity of Watson and Crick’s claim. Tautomerization is a phenomenon of structural isomerism not just exclusive to spontaneous mutagenesis but observed in many compounds across organic chemistry (for example, in biological enzymes^2^) that occurs via proton transfer mechanisms. For conciseness, we take tautomerization to mean DNA base pair tautomerization in this paper. The process of tautomerization proceeds as follows. Each hydrogen bond between the canonical base pairs of DNA depends on the bonding strength of a hydrogen atom to the more electronegative atom on the opposite base (either nitrogen or oxygen). This hydrogen atom is preferentially associated with its donor base, but via tunnelling or a classical over-the-barrier hopping mechanism, the proton (of the hydrogen atom) may travel along a minimum energy pathway to associate with the acceptor of the opposite base, leaving its electron with the base it was initially covalently bonded to. In this case, each base becomes charged, and the product is a zwitterionic base pair. This mechanism can be described as A-T ↔ A^+^-T^–^ and G-C ↔ G^–^-C^+^. It is also possible that this initial proton transfer will prompt the transfer of a second proton belonging to the other base, with the final product being two neutral bases, each with misplaced hydrogen atoms (see Figure 1). This double proton transfer is known as tautomerization, and each tautomeric base is known as a tautomer. The overall tautomerization mechanism can be written: A-T ↔ A*-T*, G-C ↔ G*-C* (where “*” denotes a tautomer). If the tautomeric form is established when mitosis begins, the tautomerized base pair will be cleaved, preventing each base pair from reverting to its respective canonical form. See Figure 1 for an illustration of the chemical reaction of tautomerization for the A-T base pair.

A tautomer can bond in the following pairs (defined by their molecular geometries): A*-C, A-C*, G*-T, and G-T*.^3^ Due to the geometric similarity between purine-pyrimidine bonding within both canonical and tautomeric pairings, the tautomer will not disrupt the replication process. The tautomeric mismatch can evade correction by the replisome fidelity checks, and a genetic error can be established in two DNA double helices, propagating through subsequent generations of DNA replication. It is also possible for the zwitterionic products of single proton transfer to bond in an *anti-syn* mismatch (where a base bonds in a flipped orientation) to form nonstandard base pair configurations.^4^ However, these anti-syn products are energetically unfavorable and cannot be incorporated into a full DNA sequence due to sterical repulsion.^5,6^ Therefore, a single proton transfer cannot propagate as a genetic mutation since it will interrupt the process of mitosis and be corrected.

To be of any relevance for genetic mutation, the products of tautomerization must survive the mitotic replication of DNA. The replication includes the cleaving of the DNA duplex, caused by the enzyme helicase forcing itself between the two strands of DNA and splitting the base pairs that bind the double helix together. The absence of the A-T tautomer in the equilibrium state of DNA, where the double helix is unperturbed by external forces pulling the structure apart, has been well-studied,^7−10^ but the existence of tautomers on single, separated DNA strands have not been confirmed. Florian and Leszczyński^7^ state that the tautomers must outlive the strand separation time scale of ∼100 ps. However, multiple studies have argued that the tautomeric lifetime is much shorter than this time scale.^11,12^ The time scale for which the tautomeric forms of base pairs can exist depends on the stability of the energetic minimum that the tautomeric forms exhibit.

Several computational studies have attempted to measure the minimum energy pathway of the tautomerization reaction in both A-T and G-C base pairs. These studies have been detailed in the reviews by Kim et al.^13^ and Srivastava^12^ but vary in methodology and, consequently, have produced different conclusions about the stability of the A*-T* tautomeric base pair. Some studies suggest the configuration is metastable,^14,15^ some suggest that there is a shallow energy minimum,^16^ and others suggest that there exists a substantial energy minimum which is sufficiently stable to be considered a candidate for the spontaneous mutagenesis mechanism.^17^

In response to the disagreement classifying the nature of A*-T* stability, Brovarets’ et al.^9^ employed Møller–Plesset second-order perturbation theory (MP2) with the 6-311++G(d,p) basis set to model the double proton transfer tautomerization in both A-T and G-C base pairs. The author reported the lifetimes of the A*-T* and G*-C* base pairs as 6.5 and 160 fs, respectively, revealing that the A*-T* configuration could not exist long enough in equilibrium to be relevant to spontaneous mutagenesis.

To reduce the computational cost of the simulations while retaining much accuracy, Soler-Polo et al.^8^ implemented a quantum mechanical/molecular mechanical (QM/MM) approach where the main system (the base pair) was computationally modeled with quantum mechanical calculations and a surrounding environment of the DNA double helix, and the aqueous solution was modeled with classical molecular mechanical calculations. This study found that the transition state of the G-C tautomerization is asymmetric; the state G*-C* is less stable than the state G-C.

A study of the tautomerization process in both A-T and G-C base pairs was conducted by Slocombe et al.^10^ and confirmed the transition state calculations in the G-C base pair of Soler-Polo. Using density functional theory (DFT), Slocombe et al. were able to discern the energy barriers of the G-C and A-T base pairs. It was discovered that the G-C base pair allows a stable minimum for the canonical and tautomerization configurations with the same asymmetry of the transition state as Soler-Polo. However, the A-T base pair, while having a stable canonical configuration, has only a metastable tautomeric configuration, in agreement with multiple prior studies.

A limitation in each of the studies above is that calculations were performed on the DNA structure in equilibrium, where the strands are not undergoing separation as they would during replication. However, as proposed by Florian and Leszczynski,^7^ the tautomeric state must be stable enough to survive the DNA cleavage within the mitosis scheme; hence, the separation of DNA strands must be considered in order to validate proton transfer between bases as a genetic mutation mechanism.

In a recent letter by Winokan et al.,^18^ tautomerization of the G-C base pair while undergoing DNA strand separation was investigated using DFT and molecular dynamics (MD) approaches. The conclusions of the DFT investigations determined that the tautomerization energy barrier increases as the strands move away from one another, as would be expected. The motion of the bases within this process was also given rotation degrees of freedom around a fixed atom of the base bonded to the sugar–phosphate backbone. It was determined that the hydrogen bonds between the bases extended quasi-linearly during DNA strand separation and that the bases rotate to preferentially limit the extension of the upper and lower, but not central, hydrogen bonds between the G-C base pair. Additionally, the MD investigation provided estimations for the time scale of DNA stand separation. The authors concluded that the lifetime of the tautomeric state must outlive 1.7 ps—a speed 2 orders of magnitude smaller than quoted by Florian and Leszczynski.^7^

**Figure 2:**
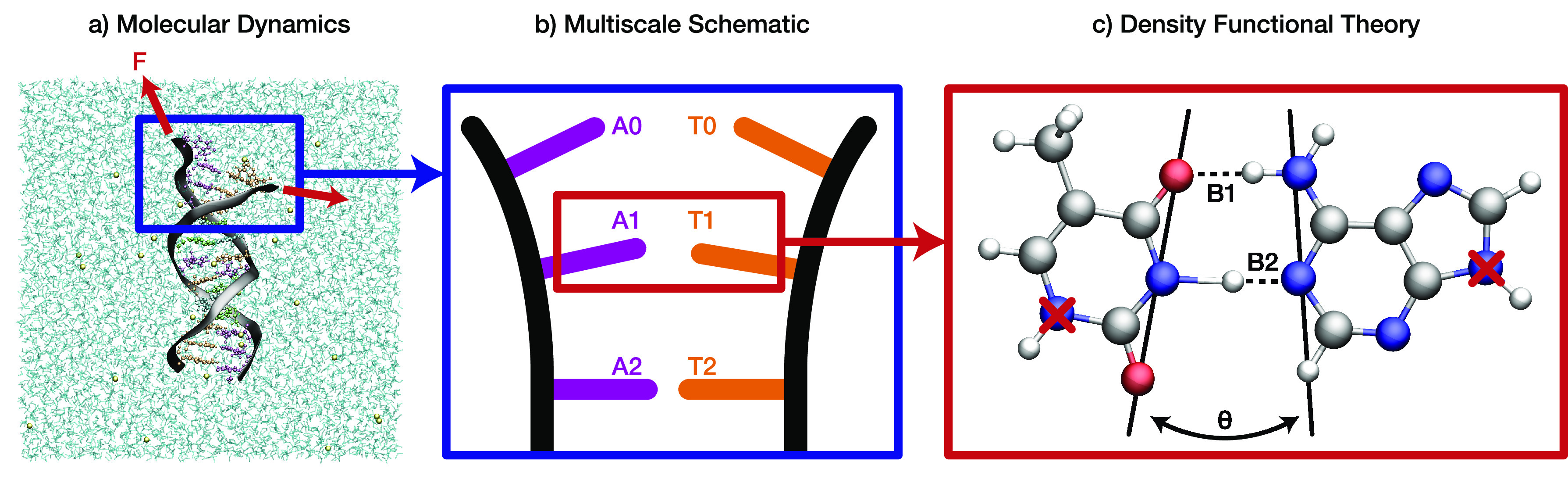
Scheme modeling DNA strand separation for the canonical form of the adenine-thymine base pair. In the MD simulation (a), the two DNA strands are forced apart by a constant steering force mimicking the action of helicase.^33^ The base pairs A0-T0 and A2-T2 are superfluous to the DFT calculations, as were the sugar–phosphate backbone strands, which are both contained within the molecular mechanical region. DFT calculations were applied to the quantum mechanical region of the A1-T1 base pair (c) and modeled the extension of the two hydrogen bonds B1 and B2 and the transition state of an asynchronous double proton transfer reaction along the bonds B1 and B2 with an intermediate state of A1^+^-T1^–^. A1 and T1 comprise the chemical formula C_10_H_11_N_7_O_2_. The A1-T1 bond opens under this action at an angle of θ. The nitrogen atoms with lock icons were fixed to the sugar–phosphate DNA backbone and served as the point around which the bases could rotate.

In this paper, we model the tautomerization of A-T during DNA strand separation. To do this, we use DFT to calculate the stretching of the hydrogen bonds B1 and B2 and between A-T and the opening angle, θ, of the base separation (see Figure 2) as a function of strand separation distance and compute the transition states of the tautomerization reaction across a range of strand separations. MD was employed to investigate the dynamics of A-T tautomerization and estimate the time scale of the reaction. A detailed description of the methods used in this investigation can be found in the Supporting Information document. The MD trajectories reveal a significant variation in the separation dynamics with profound physical implications for the tautomerization of A-T.

## Method

The DNA strand separation process is modeled by density functional theory (DFT) at the B3LYP+XDM/6-311++G** ^19,20^ level of theory. The software conducting this method is NWChem 7.0.2.^21^ We select the combination of B3LYP^19^ (exchange–correlation functional) with XDM^22,23^ (nonempirical dispersion scheme) and 6-311++G** (basis set incorporating dispersion and polarization corrections) to satisfy accuracy requirements while allowing for a reasonable computational cost. The precedent for this combination of factors comes from a recent study by Gheorghiu,^24^ who has optimized the combination of methodological approaches to attain the level of accuracy required in the multiscale modeling of DNA processes. The DNA system that the calculations pertain to is a single A-T base pair (depicted by A1-T1 in the scheme of Figure 2) which exists within an implicit continuum solvation model^25−27^ with a low dielectric factor (ϵ = 8.0).^28,29^ The low dielectric factor is in concordance with the proximity of helicase and the aqueous solution of water molecules in which the system is embedded.

We impose nonequilibrium DNA strand separation across 13 systematic increments. The separation of the strands is measured by the summed distance that the R-groups are displaced from equilibrium. The optimization of the atomic geometries within the base pairs was conducted using the L-BFGS method.^30^ We use the Atomic Simulation Environment (ASE)^31,32^ to implement the L-BFGS algorithm in order to optimize the atomic geometries and allow the system to “relax” with a force tolerance of 0.01 eV Å^–1^. During the relaxation, we fix the location of the R-group of the base to have control over the definition of the DNA strand separation. The bases are also allowed to rotate about the fixed nitrogen atoms.

We calculate the transition state of the proton transfer reactions from the canonical configuration to the tautomeric configuration of the A-T base pair across 15 snapshot images for the first eight increments of the DNA strand separation (from 0.0 to 1.56 Å separation) using a machine learning nudged elastic band (ML-NEB)^34,35^ approach. ML-NEB seeks to minimize the energy pathway of the reaction to obtain the transition state for each separation. The bases are permitted to rotate around the fixed nitrogen atoms. In this way, the bonds B1 and B2 can extend with the DNA strand separation, but not necessarily at equal rates since the bases rotate individually, limiting the extension of one bond and accelerating the extension of the other. This feature is measured by obtaining the respective bond stretching for B1 and B2 as a function of DNA strand separation. We compute the reaction asymmetry to demonstrate the asynchronicity of the reaction pathway. The ML-NEB algorithm’s advantage is minimizing the number of single-point DFT calculations to determine the energy and forces to obtain a minimum reaction energy path. An implicit assumption in transition state theory that we implement is that the reaction pathway obtained is a one-dimensional potential energy surface; therefore, the proton transfer occurs across a single dimension (the reaction coordinate along the minimum energy path) as a first approximation. ML-NEB utilizes a Gaussian regression model to reconstitute the full minimum energy path. This renewed minimum energy path is described as a surrogate minimum energy path. This surrogate minimum energy path’s convergence criteria are derived from the data points’ uncertainty along its energy pathway. The reaction path is calculated and optimized with a force tolerance of 0.01 eV Å^–1^ and a maximum energy uncertainty of 0.02 eV. Mathematically, we define the reaction path as the magnitude of two vectors: the difference between the vectors between the hydrogen donor and hydrogen acceptor in bonds B1 and B2. Throughout this investigation, the NWChem software was connected to Python3 via ASE.

We use molecular dynamics (MD) to investigate the details of the DNA strand separation process. These calculations were performed in Gromacs version 2018^36^ with the CHARMM36 (March 2019) force field^37^ and SPCE water model.^38^ The MD system is constructed of 14 base pairs within a double strand of DNA, initially in equilibrium and an aqueous environment. For each replica simulation, the system is minimized to 12 kJ mol^–1^ nm^–1^, before being placed in an *NVT* ensemble at 310 K where a 500 ps equilibration is conducted with a 1 fs time step. Following successful equilibration, a production 200 ps MD trajectory is simulated with an optional constant steering force between the backbones of the terminal A-T base pair of the DNA duplex. The steering force, if present, was applied along the vector connecting the centers of mass of the nucleotide’s backbone atoms with the force encouraging their separation. In addition to replicas where no force was applied, five other steering forces were investigated ranging from 25 to 125 kJ mol^–1^ nm^–1^. The strength of the biasing force was chosen to be of the same order of magnitude as the hydrogen bonds binding the A-T pair. In total, over 34 ns of MD trajectories across six force constants and 49 replicas were analyzed.

We calculate the occurrences of the opening angles θ (see Figure 2) across the range of DNA strand separation 0.0–2.0 Å and estimate the speed at which the strand separation occurs. In the quantum mechanical investigations, the opening angle smoothly increases during strand separation. In the MD calculations, we examine whether this observation remains true in the biological ensemble. We study the opening angle in two scenarios: where the B1 bond is the first to open and where the B2 bond is the first to open. We collect a histogram of opening angles and their occurrences across a 75° range for each scenario of B1 and B2 opening first. We treat a positive angle as opening from the B2 end of the base pair and a negative angle as opening from the B1 end of the base pair.

In both DFT and MD calculations, we simulate an environment contained within a dielectric solvent shell. Therefore, the environmental interaction with the base pair system is consistent between both methodologies.

## Results

**Figure 3:**
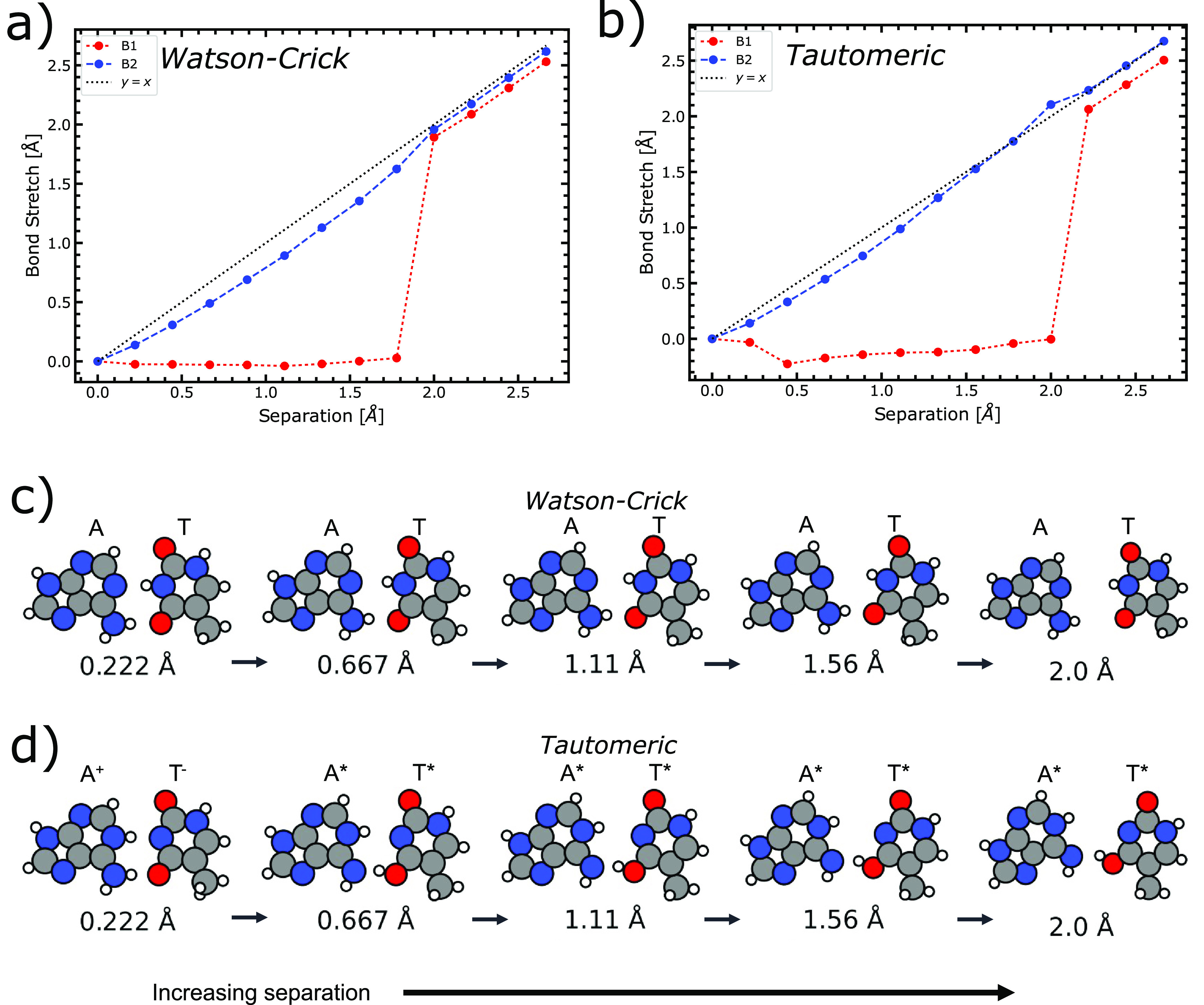
Results of the DFT model compute the scheme by which the base pair A1-T1 (see Figure 2) separates in relation to the base rotation and the stretching of the hydrogen bonds. (a and b) The stretching of the bonds B1 and B2 in the A1-T1 base pair as a function of the DNA strand separation distance for both the (a) Watson–Crick canonical configuration of the A1-T1 base pair and the (b) tautomeric A1-T1 base pair. (c and d) A five-step incremental molecular depiction of the A-T base pair under DNA strand separation for (c) the Watson–Crick canonical configuration and (d) the tautomeric configuration. The numerical labels, in angstroms, represent the distance separation of the DNA strands. In this regime, the distance 0.0 Å represents the DNA strands in equilibrium separation. The right-hand arrows define the progression of the strand separation. In the first image of panel (d), there is no stable double proton transfer mechanism for tautomerization, and the product of single proton transfer is zwitterionic, hence the labels “A^+^” and “T^–^”.

**Figure 4:**
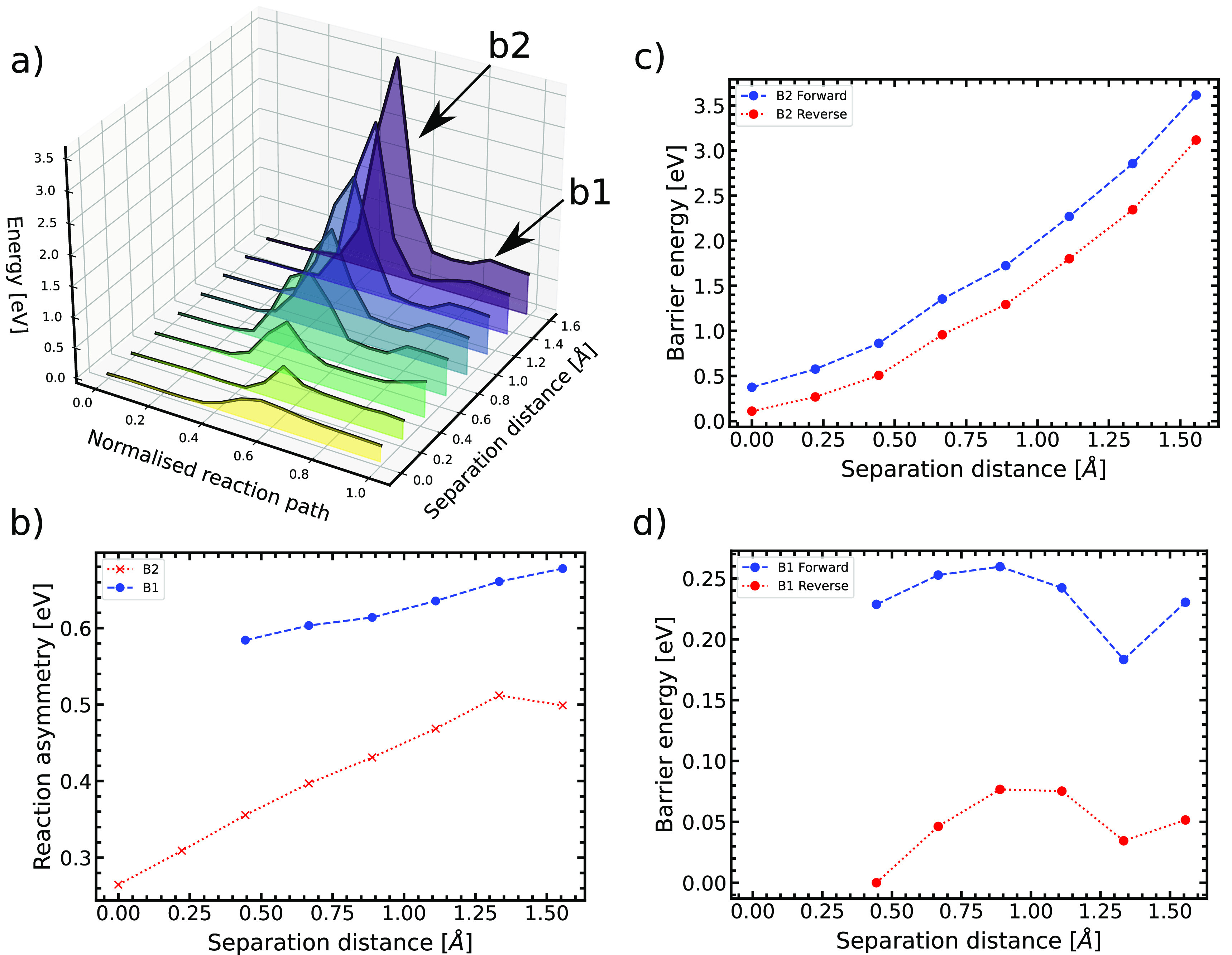
(a) The minimum energy pathway of the adenine-thymine tautomerization reaction as a function of normalized reaction path and DNA strand separation distance. Double proton transfer becomes stable at the third DNA strand separation increment, although this is difficult to see on the plot. (b) The reaction asymmetry defines the difference between the energy of the product (tautomeric state) and the energy of the reactant (canonical state). There is a single proton transfer product for all data points and a double proton transfer product after the third data separation point. (c) and (d) The reaction energy barrier between the forward and backward tautomerization reaction is plotted as a function of separation distance for both single and double proton transfer tautomerization. The two proton transfer events along bonds B1 and B2 are asynchronous.

The results of the DFT investigation are composed of data that are each a function of DNA strand separation: hydrogen bond stretching (Figure 3), the A-T tautomerization transition state (Figure 4a), the reaction asymmetry (Figure 4b), and the forward and reverse energy barriers of each concerted proton transfer (Figure 4c).

**Figure 5:**
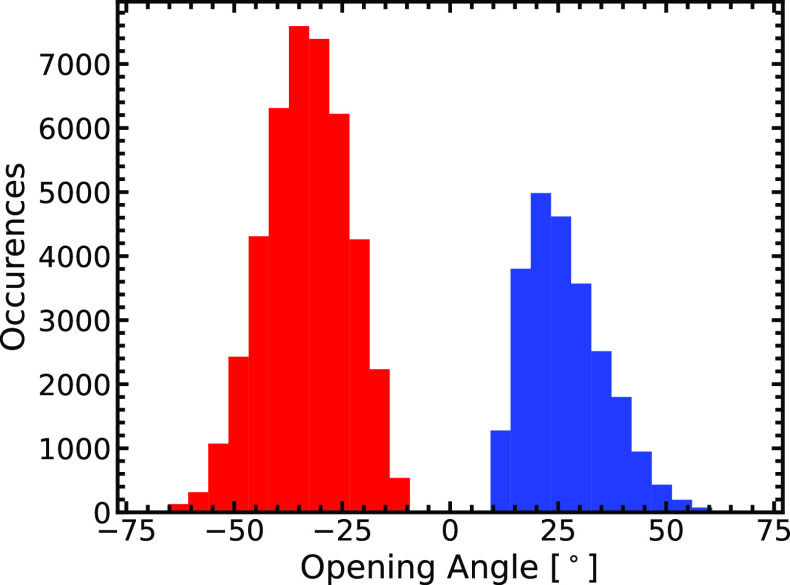
Histogram of opening angles against the occurrences corresponding to them across the range of DNA strand separation represents the energetic preferences of each opening angle summed across the range of DNA separation. The positive angles represent the opening of the base pair starting at bond B2, and the negative angles represent the opening of the base pair starting at bond B1.

**Figure 6:**
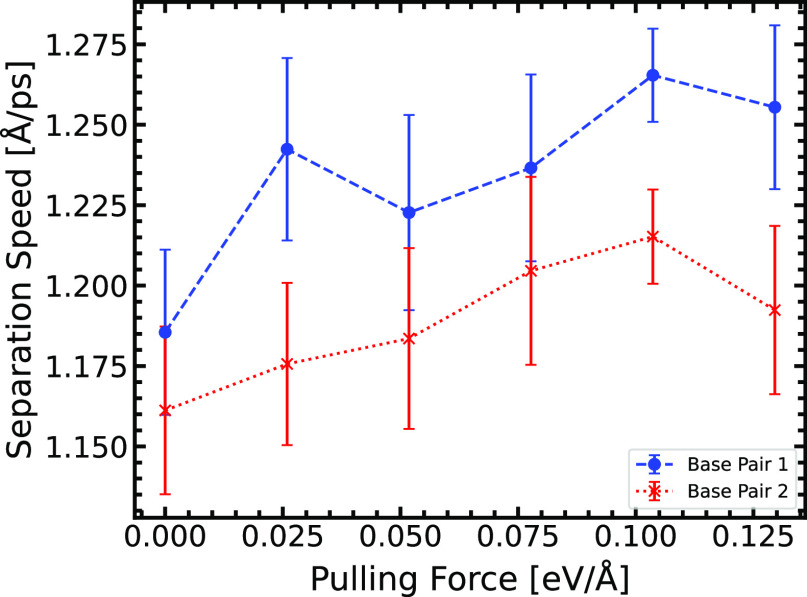
DNA strand separation speed instigated by the simulated helicase pulling force. Base Pair 1 refers to the base pair A1-T1, and Base Pair 2 refers to the base pair A2-T2 (see Figure 2). The separation speed data points provided are the arithmetic mean of 210 MD simulations (with the standard error providing the error bars) produced for six simulated helicase pulling forces.

The results of the MD investigation consist of a histogram of the statistical ensemble of angle occurrences (Figure 5) and the separation speeds of the DNA strands for each simulated helicase pulling force (Figure 6).

## Discussion

In both the canonical and tautomeric configurations of A-T, the bond extension under strand separation (see Figure 3a,b) is comparable for both individual cases of the extension of the two hydrogen bonds holding the base pair together, defined (see Figure 2) as B1 and B2. Bond B1 extends at a much more gradual rate than B2, showing that the bases are rotating with respect to one another. In both canonical and tautomeric cases, the rotation of the bases preferentially limits the extension of the B1 bond. We observe that bond B2 effectively stretches at the same rate at which the DNA strands are separated. The implication of this is that the bond B1 is the stronger bond of the two. Another similarity between the canonical and tautomeric configurations is that the opening angle between the base pair grows steadily until a cutoff point (which is different for each configuration) where the angular interaction of the bases reverts to approximately the original respective opening angle, where θ = 0. This reversion occurs at a lower separation distance for the canonical configuration (∼1.8 Å) than for the tautomeric configuration (∼2.0 Å). By establishing that the bond B1 appears to be stronger than B2, one can go further to say that the tautomeric configuration of the bond B1 is stronger. Figure 3b confirms that the tautomeric version of B1 is stronger since, initially, the bond shortens, momentarily forcing an increase in the rate of angular opening. Once the angular reversions visible in Figure 3a,b have occurred, both bonds extend in both the canonical and tautomeric configurations at a very similar rate. This rate is similar to the rate of strand separation.

In Figure 3c,d, the molecular structures of a subset of the strand separation mechanism are presented. One can observe the gradual increase in θ complementing the bond stretching patterns visible in Figure 3a,b. Two key features in Figure 3c,d are the last image of the Watson–Crick sequence and the first image of the tautomeric sequence. In the last Watson–Crick image (at 2.0 Å separation), the angle of base separation has reverted to θ = 0; the bond stretching is no longer asymmetric. This reversion to roughly the equilibrium angle is observed in Figure 3a. The first tautomeric image represents only a single proton transfer; hence, the product is a zwitterionic base pair. In the first two data points (at a strand separation of 0.0 and 0.22 Å), there is no stable energy minimum for the double proton transfer tautomeric form. Therefore, for the A*-T* configuration to be biologically relevant as a mutation mechanism, the DNA strands must first be separated by some distance as a criterion of spontaneous mutagenesis. Beyond this paradigm-shifting distance, the tautomeric configuration becomes more and more stable due to the potential energy surface having an increasing energy barrier. However, this energy barrier increase also requires greater energy to overcome. Therefore, we can conclude that the tautomeric form A*-T* becomes more stable under DNA separation but at the cost of the double proton transfer reaction becoming more unlikely.

Figure 4a represents the previous conclusion: the energy barrier between the canonical and tautomeric state grows as the distance between the DNA strands increases. However, while there is no energy minimum at the maximum coordinate on the normalized reaction path axis for the first two separation increments, an energy minimum becomes visible for larger separation distances, representing a stable state for the A*-T* tautomer. Also, in Figure 4a, one can observe the development of a central energy minimum for the third separation increment onward. This energy minimum is created by the intermediate zwitterionic state. We observe that the energetically preferable double proton transfer mechanism is asynchronous (a stepwise transfer mechanism) and consists of a single proton transfer from the thymine base to create the zwitterionic intermediate A^+^-T^–^ followed by an induced, second single proton transfer from the adenine base. The height of the second energy barrier is much lower than the first (across all strand separations and increasingly so as strand separation increases), showing that it is much more energetically favorable for the zwitterionic product to undergo single proton transfer again to produce the A*-T* tautomeric state, rather than a single proton transfer which reverts to the canonical state.

Figure 4b shows the quasi-linear reaction asymmetry of both the single and double proton transfer reactions. The reaction asymmetry depicts the variation in the stability of both the single and double proton transfer products in relation to the canonical state. One observes that the canonical state is the most stable. Figure 4b shows that the base pair structure at each increment has access to a single proton transfer minimum, but this is not the case for the double proton transfer, which can only begin to obtain stability of the double proton transfer tautomer at ≥0.444 Å strand separation (the third imposed strand separation increment).

Figure 4 panels c and d represent the change in barrier heights for transfer as a function of separation distance. The double proton transfer occurs in two single proton transfer stages, initiated by a proton donation from thymine to adenine. This first stage concerns the transfer of the proton in bond B2 from the nitrogen of thymine in B2 to the nitrogen of adenine in B2 and has a higher energy barrier across all increments of the strand separation. Initially, there is no response of a second proton transfer for the first two increments of strand separation, and the single proton transfer is all that occurs. Since there is no second proton transfer up until 0.444 Å, the first two data points for both “B1 Forward” and “B1 Reverse” are 0 eV. However, the energy barriers of the second proton transfer grow in response to the gradual creation of a stable minimum for the double proton transfer tautomeric state. Since this second proton transfer is instigated by the first, it has a lower energy barrier and grows at a much slower rate across the strand separation compared to the steep linear increase of the initial single proton transfer.

There have been indications in previous literature that the tautomerization transition state is significantly affected by the environment the base pair exists within conducted by, for example, Li et al.^39^ In their study of the particular case of DNA base pair tautomerization occurring in the wobble mismatch geometry wG-T, Li et al. report that the presence of an aqueous environment or the presence of a DNA duplex makes the transition state of a wG-T → G-T* reaction (where “wG-T” indicates a “wobble” mismatch bonding) slightly more endoergic. In contrast, the presence of a DNA polymerase enzyme makes the transition state slightly more exoergic. Therefore, exact and realistic environmental modeling can either marginally stabilize or destabilize the tautomer.

For the molecular dynamics investigation, we find that the DNA strand separation occurs at a speed of ∼1.25 Å ps^–1^. If we assume that, at a separation distance of 2.0 Å, the tautomerization reaction does not reverse back to the canonical state, the lifetime of the tautomer need only exceed ∼1.6 ps. This assumption is very reasonable since, at separations >1.56 Å, the reverse energy barrier would require temperatures of >3.5 × 10^4^ K (*E* = *k*_*B*_*T*, where *k*_*B*_ is the Boltzmann constant). Such high temperatures are not biological. Therefore, the tautomers can be trapped on separated DNA strands, surviving the mitotic division, ready to incorporate errors in the genetic code through further generations of mitosis.

Figure 5 shows the bimodal statistical distribution of the opening angles, θ, for the MD simulations across the DNA strand separation range. For bond B2 opening first, the process favors an opening angle of ∼20°, showing that the most energetically stable mode by which this takes place is when this angle is observed. The second scenario shows the process favors an opening angle of ∼−35°, corresponding to the initial opening bond being B1. Across both scenarios, the most energetically favorable configuration of the DNA strand separation overall is at an angle of ∼35° with the DNA unzipping from the B1 bond (i.e., the strand in Figure 2 unzipping from the bottom upward). Figure 5 shows that favoring ∼−35° as an opening angle is universal and cumulative across the DNA separation process.

## Conclusions

Using DFT calculations, we propose that the double proton transfer tautomer of the adenine-thymine base pair (A*-T*) can exist in an energetically stable state on the condition that the DNA duplex is separated by more than 0.444 Å. As the DNA strands separate further from one another, the A*-T* state becomes more stabilized due to the increase in the energy barriers of the tautomerization reaction. However, this increasing energy barrier also diminishes the probability of the tautomerization reaction occurring. Before the separation that stabilizes the A*-T* state is reached, only single proton transfer products are stable, but these reactions are not biologically relevant to the spontaneous mutagenesis process. However, the double proton transfer product is biologically relevant to the spontaneous mutagenesis process as its tautomeric nucleobases can bond in noncanonical pairings that evade replisome fidelity checks. The conclusion that the A*-T* base pair can be a genetic mutation instigator is contrary to several previous studies^7,14,15^ which have only examined the tautomerization of A-T while the DNA duplex is in an equilibrium state, finding that the state A*-T* at equilibrium is metastable and therefore biologically irrelevant.

Using MD calculations, we find that the mode of DNA strand separation that is energetically favored is where the helicase enzyme forces open the B1 bond first at an angle of ∼35°. We also find that the minimum lifetime of the tautomer to be mechanistically feasible for genetic mutation is ∼1.6 ps. This minimum lifetime of the A*-T* base pair is 2 orders of magnitude shorter than previously suggested by Florian et al.^7^ and in agreement with the minimum lifetime of the G*-C* base pair suggested by Slocombe et al.^18^ of 1.7 ps.

**Figure 7:**
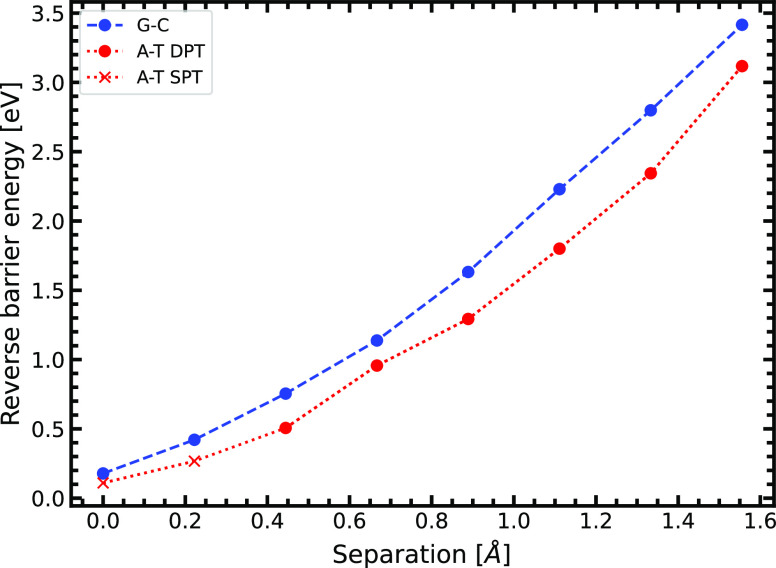
Reverse energy barrier of the first proton transfer as a function of the DNA strand separation. The A-T reverse energy barrier data are split into two portions: SPT (single proton transfer), of which the product is the zwitterionic A^+^-T^–^ state, and DPT (double proton transfer), of which the product is the tautomeric base pair state A*-T*.

A representation of the stability of the tautomers (reverse energy barrier of the tautomerization reaction) G*-C* and A*-T* is shown in Figure 7. We observe that the reverse energy barrier of both tautomers increases during strand separation at a similar rate. The relation between separation distance (*x*) and energy barrier (*y*) is close to quadratic; *y* ∝ *x*^1.894^ for A-T tautomerization and *y* ∝ *x*^1.783^ for G-C tautomerization. By asserting that the stability of the tautomeric product is associated with the height of the tautomerization energy barrier, we can say that the states G*-C* and A*-T* are very similar in energetic stability from 0.44 Å where A*-T* begins to be formed.

In this paper, we examine the mechanical effect of the separation process on A-T tautomerization facilitated by the helicase enzyme. It is possible that the helicase microenvironment might affect the reaction free energy to stabilize or destabilize the A-T tautomer. However, it still needs to be determined to what extent the helicase is simply a mechanical device that forces the DNA strands apart or whether the helicase provides a significant electrostatic interaction to alter the energy landscape of A-T tautomerization. Two points speak in favor of this paper. First, the base pair A1-T1 is enclosed in a molecular environment between A0-T0 and A2-T2, which one would expect to shield the quantum mechanical region from the electrostatic potential of the helicase enzyme. Second, the quantum mechanical calculations at equilibrium with no induced separation in Figure 4 are in excellent agreement with those of Gheorghiu et al.,^3,24^ who consider the environmental effect of water complexes in DNA proton transfer.

To the best of our knowledge, our theoretical study provides the first strong argument against the widely held belief that adenine-thymine tautomers are not relevant to spontaneous mutagenesis. By incorporating the dynamical process of DNA strand separation—a key step in the mitotic replication process and much more realistic than a static DNA picture—we show that adenine-thymine tautomerization is just as relevant in genetic mutation mechanisms as guanine-cytosine tautomerization.

## Data Availability

The data presented in the figures of this article are available from the corresponding authors upon reasonable request. The reaction pathways and structures are available on Github (https://github.com/LouieSlocombe/Effect-of-Strand-Separation-on-AT-Tautomerism).

## Supporting Information Available

Three-step description of the spontaneous mutagenesis process, possible non-standard, Watson-Crick-like base pairings, description of how the possible nonstandard Watson–Crick-like base pairings can develop due to the proton transfer, and an expanded description of the molecular dynamics simulations (PDF)

## References

[ref1] Watson J.; Crick F. Genetical Implications of the Structure of Deoxyribonucleic Acid. Nature 1953, 171, 964–967. 10.1038/171964b0. 13063483

[ref2] Nemeria N. S.; Chakraborty S.; Balakrishnan A.; Jordan F. Reaction mechanisms of thiamin diphosphate enzymes: defining states of ionization and tautomerization of the cofactor at individual steps. FEBS journal 2009, 276, 2432–3446. 10.1111/j.1742-4658.2009.06964.x. 19476485 PMC2795322

[ref3] Gheorghiu A.; Coveney P.; Arabi A. The influence of base pair tautomerism on single point mutations in aqueous DNA. Interface focus 2020, 10, 20190120 10.1098/rsfs.2019.0120. 33178413 PMC7653342

[ref4] Rozov A.; Demeshkina N.; Khusainov I.; Westhof E.; Yusupov M.; Yusupova G. Novel base-pairing interactions at the tRNA wobble position crucial for accurate reading of the genetic code. Nat. Commun. 2016, 7, 10457 10.1038/ncomms10457. 26791911 PMC4736104

[ref5] Kimsey I. J.; Szymanski E. S.; Zahurancik W. J.; Shakya A.; Xue Y.; Chu C.-C.; Sathyamoorthy B.; Suo Z.; Al-Hashimi H. M. Dynamic basis for dG• dT misincorporation via tautomerization and ionization. Nature 2018, 554, 195–201. 10.1038/nature25487. 29420478 PMC5808992

[ref6] Kryachko E. S.; Sabin J. R. Quantum chemical study of the hydrogen-bonded patterns in A· T base pair of DNA: Origins of tautomeric mispairs, base flipping, and Watson–Crick ⇒ Hoogsteen conversion. International journal of quantum chemistry 2003, 91, 695–710. 10.1002/qua.10462.

[ref7] Florian J.; Leszczyński J. Spontaneous DNA Mutations Induced by Proton Transfer in the Guanine ⊙ Cytosine Base Pairs: An Energetic Perspective. J. Am. Chem. Soc. 1996, 118, 3010–3017. 10.1021/ja951983g.

[ref8] Soler-Polo D.; Mendieta-Moreno J. I.; Trabada D. G.; Mendieta J.; Ortega J. Proton transfer in guanine-cytosine base pairs in B-DNA. J. Chem. Theory Comput. 2019, 15, 6984–6991. 10.1021/acs.jctc.9b00757. 31665604

[ref9] Brovarets’ O. O.; Hovorun D. M. Atomistic mechanisms of the double proton transfer in the H-bonded nucleobase pairs: QM/QTAIM computational lessons. J. Biomol. Struct. Dyn. 2019, 37, 1880–1907. 10.1080/07391102.2018.1467795. 29676661

[ref10] Slocombe L.; Al-Khalili J.; Sacchi M. Quantum and classical effects in DNA point mutations: Watson–Crick tautomerism in AT and GC base pairs. Phys. Chem. Chem. Phys. 2021, 23, 4141–4150. 10.1039/D0CP05781A. 33533770

[ref11] Jacquemin D.; Zuniga J.; Requena A.; Céron-Carrasco J. P. Assessing the importance of proton transfer reactions in DNA. Accounts of chemical research 2014, 47, 2467–2474. 10.1021/ar500148c. 24849375

[ref12] Srivastava R. The role of proton transfer on mutations. Frontiers in Chemistry 2019, 7, 536 10.3389/fchem.2019.00536. 31497591 PMC6712085

[ref13] Kim Y.; Bertagna F.; D’Souza E. M.; Heyes D. J.; Johannissen L. O.; Nery E. T.; Pantelias A.; Sanchez-Pedreño Jimenez A.; Slocombe L.; Spencer M. G.; et al. Quantum biology: An update and perspective. Quantum Reports 2021, 3, 80–126. 10.3390/quantum3010006.

[ref14] Gorb L.; Podolyan Y.; Dziekonski P.; Sokalski W. A.; Leszczynski J. Double-proton transfer in adenine- thymine and guanine- cytosine base pairs. A post-hartree- fock ab initio study. J. Am. Chem. Soc. 2004, 126, 10119–10129. 10.1021/ja049155n. 15303888

[ref15] Cerón-Carrasco J.; Requena A.; Zúñiga J.; Michaux C.; Perpète E.; Jacquemin D. Intermolecular proton transfer in microhydrated guanine- cytosine base pairs: A new mechanism for spontaneous mutation in DNA. J. Phys. Chem. A 2009, 113, 10549–10556. 10.1021/jp906551f. 19736955

[ref16] Villani G. Theoretical investigation of hydrogen transfer mechanism in the adenine–thymine base pair. Chemical physics 2005, 316, 1–8. 10.1016/j.chemphys.2005.04.030. 20200744

[ref17] Villani G. Theoretical investigation of hydrogen atom transfer in the adenine–thymine base pair and its coupling with the electronic rearrangement. Concerted vs. stepwise mechanism. Phys. Chem. Chem. Phys. 2010, 12, 2664–2669. 10.1039/b917672a. 20200744

[ref18] Slocombe L.; Winokan M.; Al-Khalili J.; Sacchi M. Proton transfer during DNA strand separation as a source of mutagenic guanine-cytosine tautomers. Communications Chemistry 2022, 5, 144 10.1038/s42004-022-00760-x. 36697962 PMC9814255

[ref19] Becke A. Density-functional thermochemistry. III. The role of exact exchange (1993) J. Chem. Phys. 1993, 98, 5648 10.1063/1.464913.

[ref20] Johnson E. R.; Becke A. D. Van der Waals interactions from the exchange hole dipole moment: application to bio-organic benchmark systems. Chemical physics letters 2006, 432, 600–603. 10.1016/j.cplett.2006.10.094.

[ref21] Apra E.; Bylaska E. J.; De Jong W. A.; Govind N.; Kowalski K.; Straatsma T. P.; Valiev M.; van Dam H. J.; Alexeev Y.; Anchell J.; et al. NWChem: Past, present, and future. J. Chem. Phys. 2020, 152, 184102 10.1063/5.0004997. 32414274

[ref22] Becke A. D.; Johnson E. R. A density-functional model of the dispersion interaction. J. Chem. Phys. 2005, 123, 154101 10.1063/1.2065267. 16252936

[ref23] Johnson E. R.; Becke A. D. A post-Hartree–Fock model of intermolecular interactions. J. Chem. Phys. 2005, 123, 024101 10.1063/1.1949201. 16050735

[ref24] Gheorghiu A. Ensemble-based multiscale modelling of DNA base pair tautomerism in the absence and presence of external electric fields. Ph.D. thesis, UCL (University College London), 2021.

[ref25] Klamt A.; Schüürmann G. COSMO: a new approach to dielectric screening in solvents with explicit expressions for the screening energy and its gradient. J. Chem. Soc., Perkin Trans. 1993, 2, 799–805. 10.1039/P29930000799.

[ref26] York D. M.; Karplus M. A Smooth Solvation Potential Based on the Conductor-Like Screening Model. J. Phys. Chem. A 1999, 103, 11060–11079. 10.1021/jp992097l.

[ref27] Marenich A. V.; Cramer C. J.; Truhlar D. G. Universal Solvation Model Based on Solute Electron Density and on a Continuum Model of the Solvent Defined by the Bulk Dielectric Constant and Atomic Surface Tensions. J. Phys. Chem. B 2009, 113, 6378–6396. 10.1021/jp810292n. 19366259

[ref28] Pitera J. W.; Falta M.; van Gunsteren W. F. Dielectric properties of proteins from simulation: the effects of solvent, ligands, pH, and temperature. Biophysical journal 2001, 80, 2546–2555. 10.1016/S0006-3495(01)76226-1. 11371433 PMC1301444

[ref29] Li L.; Li C.; Zhang Z.; Alexov E. On the dielectric “constant” of proteins: smooth dielectric function for macromolecular modeling and its implementation in DelPhi. J. Chem. Theory Comput. 2013, 9, 2126–2136. 10.1021/ct400065j. 23585741 PMC3622359

[ref30] Payne M. C.; Teter M. P.; Allan D. C.; Arias T. A.; Joannopoulos J. D. Iterative minimization techniques for ab initio total-energy calculations - molecular-dynamics and conjugate gradients. Rev. Mod. Phys. 1992, 64, 1045–1097. 10.1103/RevModPhys.64.1045.

[ref31] Larsen A. H.; Mortensen J. J.; Blomqvist J.; et al. The atomic simulation environment—a Python library for working with atoms. J. Phys.: Condens. Matter 2017, 29, 273002 10.1088/1361-648X/aa680e. 28323250

[ref32] Bahn S. R.; Jacobsen K. W. An object-oriented scripting interface to a legacy electronic structure code. Comput. Sci. Eng. 2002, 4, 56–66. 10.1109/5992.998641.

[ref33] Yu J.; Ha T.; Schulten K. Structure-based model of the stepping motor of PcrA helicase. Biophysical journal 2006, 91, 2097–2114. 10.1529/biophysj.106.088203. 16815905 PMC1557568

[ref34] Hansen M. H.; Torres J. A. G.; Jennings P. C.; An Atomistic Machine Learning Package for Surface Science and Catalysis. arXiv:1904.00904 2019, 10.48550/arXiv.1904.00904.

[ref35] Torres J. A. G.; Jennings P. C.; Hansen M. H.; et al. Low-scaling algorithm for nudged elastic band calculations using a surrogate machine learning model. Phys. Rev. Lett. 2019, 122, 156001 10.1103/PhysRevLett.122.156001. 31050513

[ref36] Bekker H.; Berendsen H.; Dijkstra E.; Achterop S.; Vondrumen R.; van der Spoel D.; Sijbers A.; Keegstra H.; Renardus M. Gromacs-a parallel computer for molecular-dynamics simulations. PHYSICS COMPUTING '92; 4th International Conference on Computational Physics (PC 92); World Scientific, 1993; pp 252–256.

[ref37] Hart K.; Foloppe N.; Baker C. M.; Denning E. J.; Nilsson L.; MacKerell A. D. Jr. Optimization of the CHARMM additive force field for DNA: Improved treatment of the BI/BII conformational equilibrium. J. Chem. Theory Comput. 2012, 8, 348–362. 10.1021/ct200723y. 22368531 PMC3285246

[ref38] Berendsen H.; Grigera J.; Straatsma T. The missing term in effective pair potentials. J. Phys. Chem. 1987, 91, 6269–6271. 10.1021/j100308a038.

[ref39] Li P.; Rangadurai A.; Al-Hashimi H. M.; Hammes-Schiffer S. Environmental effects on guanine-thymine mispair tautomerization explored with quantum mechanical/molecular mechanical free energy simulations. J. Am. Chem. Soc. 2020, 142, 11183–11191. 10.1021/jacs.0c03774. 32459476 PMC7354846

