# Supplementary material for: Tautomerisation Mechanisms in the Adenine-Thymine Nucleobase Pair during DNA Strand Separation
Source: J Phys Chem B. 2023 Mar 20;127(19):4220–8. doi: 10.1021/acs.jpcb.2c08631 (PMC10201536; doi:10.1021/acs.jpcb.2c08631)
Supplement: Supplementary file 1 — jp2c08631_si_001.pdf [file jp2c08631_si_001.pdf]

Supporting Information: Tautomerisation Mechanisms in the Adenine-Thymine Nucleobase Pair During DNA Strand Separation

Benjamin King,[1,*] Max Winokan,[2,†] Paul Stevenson,[1,‡] Jim Al-Khalili,[1,§] Louie Slocombe,[3,¶] and Marco Sacchi[3,**]

[1]*Department of Physics, University of Surrey, Guildford, GU2 7XH, UK.*
[2]*Leverhulme Quantum Biology Doctoral Training Centre, University of Surrey, Guildford, GU2 7XH, UK.*
[3]*Department of Chemistry, University of Surrey, Guildford, GU2 7XH, UK.*
(Dated: February 16, 2023)

This resource provides supplementary information detailing the investigative procedures and results presented in the manuscript *Tautomerisation Mechanisms in the Adenine-Thymine Nucleobase Pair During DNA Strand Separation*. The data presented in the article, reaction pathways, structures, and analysis source codes are available on Github. Additional information is available from the corresponding authors upon reasonable request.

CONTENTS

Supplementary Note 1: Adenine-Thymine Tautomerisation and Non-Standard Nucleobase Pairing 2

Supplementary Note 2: Molecular Dynamics Simulations 3

Supplementary References 4

* bk00346@surrey.ac.uk
† m.winokan@surrey.ac.uk
‡ p.stevenson@surrey.ac.uk
§ j.al-khalili@surrey.ac.uk
¶ louie.slocombe@surrey.ac.uk
** m.sacchi@surrey.ac.uk

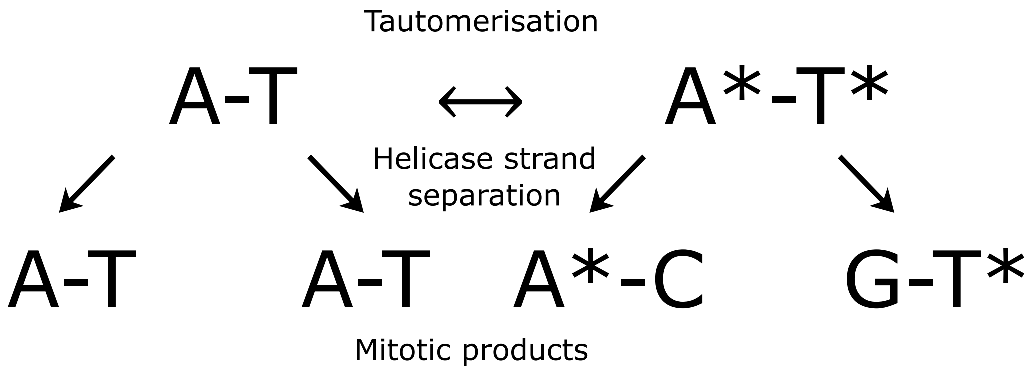

Figure S 1: A three-step description of the spontaneous mutagenesis process driven by tautomerisation of the adenine-thymine base pair.

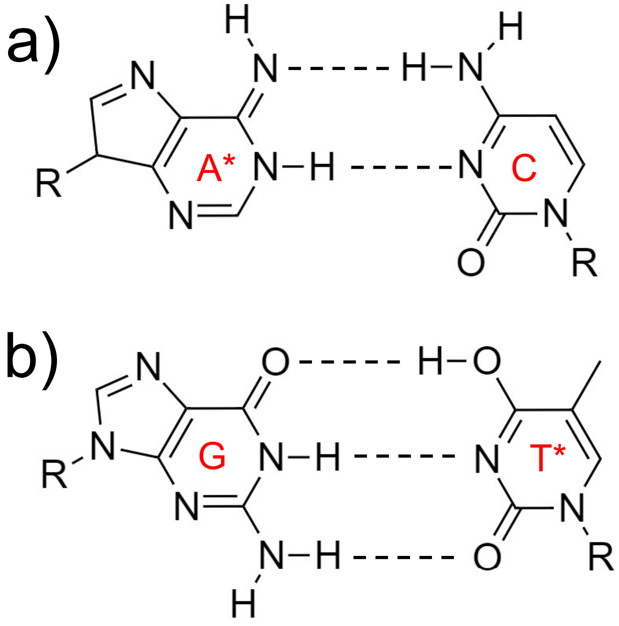

Figure S 2: The possible non-standard, Watson-Crick-like base pairings that can develop due to proton transfer. This is the incorporation of a genetic error by the tautomerisation mechanism.

SUPPLEMENTARY NOTE 1: ADENINE-THYMINE TAUTOMERISATION AND NON-STANDARD NUCLEOBASE PAIRING

The process of the tautomerisation reaction of adenine-thymine (A-T) is detailed in the manuscript. A broad overview of the tautomerisation process is presented in Supplementary Figure 1.

Once the non-standard forms of the bases, A* and T*, are established on isolated DNA strands during mitosis, the next step in the biological process is the formation of the new DNA strands by adding nucleotides at the active site of the DNA polymerase. The mutated bases A* and T* can bond in the non-standard, Watson-Crick-like pairings A*-C and G-T*. These pairings are shown in Supplementary Figure 2.

The consequence of the non-standard base pairings is that an A*-C pair is created in one strand of DNA and a G-T* pair in another instead of two A-T base pairs. These DNA code errors can evade replisome fidelity checkpoints and create equivalent errors through subsequent generations of DNA replication. The tautomerisation of A-T is an asynchronous, step-wise reaction with a zwitterionic intermediate product from a single proton transfer which

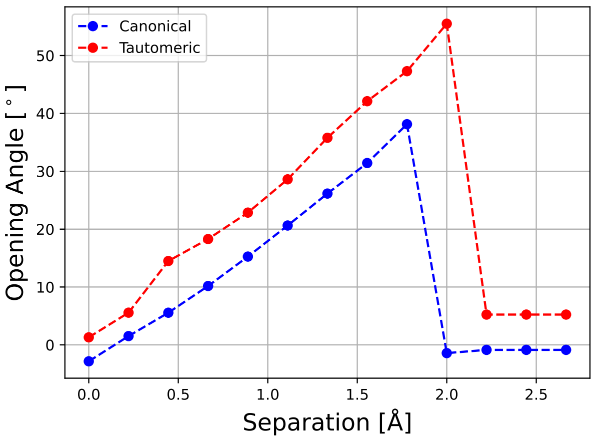

Figure S 3: The opening angles of the base pair for the canonical and tautomeric configurations across the range of DNA strand separation 0.0 Å-2.5 Å.

produces A^{+}-T^{-}. The zwitterionic bases can bond in the following pairs: A^{+}_{anti}-G_{syn}, A^{+}_{anti}-C_{syn}, and G-T^{-}_{wobble} (where 'anti' and 'syn' indicate anti-addition and syn-addition wherein the non-standard base bonding occurs upon non-standard faces of the base molecules and 'wobble' indicates a sterical bonding mismatch) [1, 2]. These base pairs are incompatible with the double helix structure, so mitosis will cease when the replication machinery encounters zwitterionic products. Therefore, only double proton transfer tautomerisation products are relevant to spontaneous mutagenesis.

SUPPLEMENTARY NOTE 2: MOLECULAR DYNAMICS SIMULATIONS

We use molecular dynamics (MD) to investigate the detail of the DNA strand separation process. We calculate the occurrences of the opening angles θ across the range of DNA strand separation 0.0 Å - 2.0 Å and estimate the speed at which the strand separation occurs. The MD system is constructed of 14 base pairs within a double strand of DNA, initially in equilibrium and an aqueous environment. In the quantum mechanical investigations, the opening angle smoothly increases during strand separation (see Supplementary Figure 3). We examine whether this observation remains true in the biological ensemble in the MD calculation. We study the opening angle in two scenarios: where the B1 bond is the first to open and where the B2 bond is the first to open. We collect a histogram of opening angles and their occurrences across a 75° range for each scenario of B1 and B2 opening first. We treat a positive angle as opening from the B2 end of the base pair and a negative angle as opening from the B1 end of the base pair.

The software we use to conduct molecular dynamics (MD) simulations is GROMACS 2018 [3]. The system consists of 14 base pairs within a DNA duplex with the base code: T^{3}TTGTACGTACAAA^{5}. A 2 nm x 2 nm x 2 nm) simulation box is constructed to surround the DNA system with explicit SPCE solvent and, neutralising sodium ions, a CHARMM36 [4] force field is employed for all the MD simulations. We minimise a group of replica systems, equilibrate them over a scale of 50 ps of NVT ensemble over incremental time steps of 1 fs and simulate 10 different separation forces with a maximum force 12 kJ mol^{-1} nm^{-1}. The system temperature is maintained, by a Nose-Hoover thermostat, at 310 K, using 0.2 ps as a coupling constant. The data is collected over 66 system replicas. We collect the time series statistics of the two hydrogen bond lengths between the adenine and thymine base pair and pass these statistics through a Savitsky-Golay filter.

We define the opening angle between the bases with the scheme presented in Supplementary Figure 4. We differentiate between positive and negative θ values by which end of the base pair opens first. An initial opening of the bond N_T-H_T—N_A (bond B2) is defined by a positive angle, and an initial opening of the bond O_T—H_A-N_A (bond B1) is defined by a negative angle.

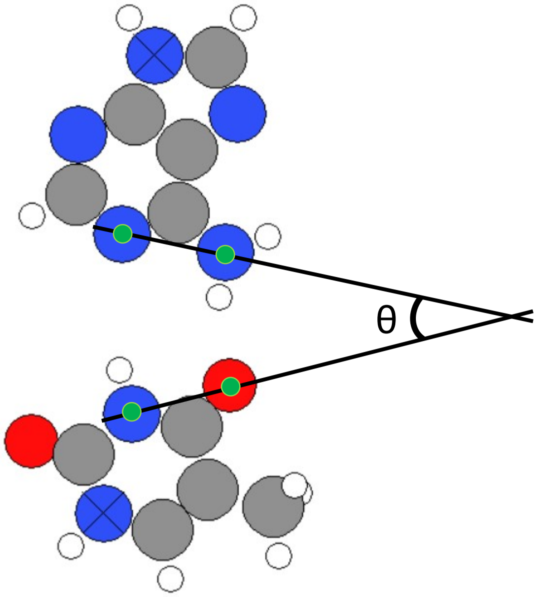

Figure S 4: The scheme by which the base pair's opening angle, θ, is determined. The angle is the dot product of the 3-dimensional vectors that pass through the hydrogen donor and acceptor atoms, indicated with green spots.

SUPPLEMENTARY REFERENCES

[1] A. Gheorghiu, P. Coveney, and A. Arabi, The influence of base pair tautomerism on single point mutations in aqueous DNA, Interface focus **10**, 20190120 (2020).

[2] Y. Kim, F. Bertagna, E. M. D'Souza, D. J. Heyes, L. O. Johannissen, E. T. Nery, A. Pantelias, A. Sanchez-Pedreño Jimenez, L. Slocombe, M. G. Spencer, *et al.*, Quantum biology: An update and perspective, Quantum Reports **3**, 80 (2021).

[3] H. Berendsen, D. van der Spoel, and R. van Drunen, Gromacs: A message-passing parallel molecular dynamics implementation, Computer Physics Communications **91**, 43 (1995).

[4] K. Hart, N. Foloppe, C. M. Baker, E. J. Denning, L. Nilsson, and A. D. MacKerell Jr, Optimization of the charmm additive force field for dna: Improved treatment of the bi/bii conformational equilibrium, Journal of chemical theory and computation **8**, 348 (2012).
